# A matter of participation? A critical incident study of municipal care personnel in situations involving care-dependent older persons and emergency medical services

**DOI:** 10.1080/17482631.2022.2082062

**Published:** 2022-06-15

**Authors:** Anna Hjalmarsson, Gunnel Östlund, Margareta Asp, Birgitta Kerstis, Mats Holmberg

**Affiliations:** a School of Health, Care and Social Welfare, Mälardalen University, Eskilstuna Västerås, Sweden; b Faculty of Health and Life Sciences, Linneaus University, Växjö, Sweden; c Centre for Clinical Research Sörmland, Uppsala University, Eskilstuna, Sweden; d Department of Ambulance Services, Region Sörmland, Eskilstuna, Sweden

**Keywords:** Ambulance dispatch personnel, ambulance personnel, critical incident technique, emergency medical services, emergency situations, experiences, inter-organizational collaboration, municipal care personnel, municipal home care services, older persons

## Abstract

**Purpose:**

This study aimed at describing municipal care personnel’s experiences of and actions in situations when older persons need emergency medical services (EMS) at home.

**Methods:**

An inductive descriptive design adhering to critical incident technique (CIT) was used. Data were collected through interviews and free text written questionnaires, analysed in accordance with CIT procedure.

**Results:**

Experiences related to the main areas of *Lifesaving competence* and *Collaborative care*. Lifesaving competence involved having sufficient knowledge to guide older persons in emergencies without organizational support. The lack of care alternatives carries dependence on inter-organizational collaboration, as well as having to accept the collaborative conditions provided by the EMS. Actions meant *Adjusting to situational needs and EMS authority*, which involved safeguarding the person while being directed by the EMS.

**Conclusions:**

Lack of organizational support, care alternatives, and structured collaboration jeopardize care-dependent older persons’ health, and ability to influence care when emergency situations occur at home. Municipal care personnel’s actions as the older person’s representative support human agency, allowing older persons to become active participants in care despite acute suffering. This study underlines the importance of further developing welfare policies that facilitate and regulate inter-organizational responsibilities of health and social care to favour older people.

## Introduction

Governments all over the world promote the benefits of older people with different and sometimes complex care needs continuing to live their lives in their own homes. The ageing in place strategy not only facilitates sound welfare economies, but is also considered to promote the independence and quality of life of older people (Rostgaard et al., 2011; Vanleerberghe et al., 2017). Complex care needs result from conditions such as multiple chronic diseases which often include both mental health and physical components (Barnett et al., 2012), physical and cognitive frailty (Collard et al., 2012; Sugimoto et al., 2018), and functional decline (Kojima, 2017; Williams & Egede, 2016). Another aspect that increases this complexity is loneliness in older age. Loneliness often relates to widowhood and cognitive and functional decline (De Koning et al., 2017), known to negatively affect health outcomes (Shankar et al., 2017) and quality of life (Vanleerberghe et al., 2017). To meet the unique care needs of persons ageing in place, health and social care organizations must support person-centred care practices (Kuluski et al., 2017) where the goal of care is meaningful life (Håkansson Eklund et al., 2019), and participation in care is key (Christensen & Pilling, 2019; World Health Organization, 2016).

The experience of quality in life when ageing in place is connected to the older person’s ability to control life, which is also found integral to health (Ebrahimi et al., 2012; Sixsmith et al., 2014). Frail older persons describe health as the ability to experience harmony and balance in life, which involves being content with life, being involved, and being seen as valuable and competent (Ebrahimi et al., 2012). When losing one’s capabilities, trusting the care provider and arranging care based on personal preferences becomes important (Claassens et al., 2014). In cognitive impairments, such as dementia, the care provider even becomes a physical and psychological necessity, underlining the importance of a person-centred care that safeguards identity, personal beliefs and values (Fazio et al., 2018). However, research indicates that being dependent on others for everyday life requires an ordering of life according to organizational prerequisites and the competence of care providers (Jarling et al., 2018). Such ordering of life means having to accept the conditions for care, despite the fact that they are not aligned to personal preferences and needs (Ploeg et al., 2019; Roin, 2018).

The forced relations characteristic of home care services are positively experienced by older persons if the care provider manages to create a caring relationship (Jarling et al., 2018), with an emphasis on preserving dignity (Witsø et al., 2015). Building such relationships underpin the care providers’ ability to listen to and value the older person’s story, and to acknowledge the whole person (Slettebø et al., 2012). Care actions are to make a difference in the older persons’ lives (Eldh et al., 2016). However, Swedish care personnel describe the caring responsibility as being limitless, and that time constraints stipulate care rather than a person’s needs (Jarling et al., 2020).

Older age and complex chronic conditions also mean an increased need of ambulance care and hospital admission (Jones et al., 2017; Shenvi & Platts-Mills, 2019). Approximately 50% of all persons aged 65–74 years in Sweden live with at least two chronic diseases, rising to 80% among people aged > 85 years (SOU 2018:39). A recent study displays that most emergency ambulance assignments to homes in Sweden involve persons aged 70 years or older (Hjalmarsson et al., 2020). Out of hospital emergencies trigger a chain of events that in Sweden involve the regionally governed emergency medical services (EMS). The EMS organization includes emergency medical dispatch centres (EMDC), and the ambulance services. The EMDC respond to all emergency calls (112, the Swedish emergency number) and allocate ambulance resources, and the ambulance services provide advanced care on-scene usually using specialist educated registered nurses (Lindström et al., 2015). Patients’ experiences of ambulance care similarly elucidate a dependence, described as a positive or negative surrendering of life into the hands of the ambulance personnel (Holmberg et al., 2014). Being considered important and taken seriously without being exposed to judgemental attitudes contributes to positive emergency experiences (Ferguson et al., 2019; Holmberg et al., 2014; Rantala et al., 2018).

The Swedish municipal home care service organization relies on transitional care in situations when older persons suffer an acute illness at home. Research focusing on acute transitional care involving EMS responses for older persons living in nursing homes states that personnel’s decisions to call for ambulances are influenced by the availability of resources and older persons’ preferences (Trahan et al., 2016), in addition to lack of time, not knowing the person, and educational level (Kirsebom et al., 2017). Transitional care for older persons in non-acute contexts also displays deficits related to communication, unclear responsibilities, and organizational constraints (Toscan et al., 2012). The governmental strategy of ageing in place makes older persons’ private homes a growing arena not only for everyday care, but also for acute care. Thus, research focusing on prehospital emergency care in relation to municipal home care is urgently required to ensure quality in care for older persons experiencing acute care needs at home. This study seeks to enhance the knowledge about out of hospital emergencies for care-dependent older persons living at home from the perspectives of municipal care personnel.

## Aim

The aim was to describe municipal care personnel’s experiences of and actions in situations when older persons need emergency medical services at home.

## Method

### Study design

The present study had an inductive descriptive design adhering to critical incident technique (CIT) as described by Fridlund et al. (2017). CIT focuses on how people experience critical situations and how those situations are handled. In researching lived experience, the method is rooted in phenomenology (Fridlund et al., 2017) and of multidisciplinary use. In relation to health care, CIT is often used in emergency settings (Benneck & Bremer, 2019; Bohström et al., 2017; Danielis et al., 2018).

The critical incident in the present study was defined as a situation when a care-dependent older person needed EMS at home. The situation was initiated by the older person pressing the mobile safety alarm, or by care personnel identifying an emergency need in contact with the older person. The situation ended when the emergency need had been responded to. The criticality of the situation is the care personnel having to face and respond to an emergency need with direct consequence for the older person’s health and well-being. International research often refers to the concept *emergency* as acute life-threatening conditions (Nehme et al., 2016; Sanko et al., 2020; Villani et al., 2017), though the concept is not predefined as such in this study. What constituted an emergency was thus left for the care personnel to define based on their lived experience of the situations.

### Study setting

The municipality under study is in the middle part of Sweden, and about 16 000 persons in the municipality are aged 70 years or older (Statistics Sweden, 2020). Of those, about 10% (*n* = 1 499) utilize home care services, and about 16% (*n* = 2 457) utilize mobile safety alarms (statistics provided by the municipality, 2021–03-31). Municipal home care services are diurnal services embracing planned and unplanned assistive responses. In the current municipality, planned home care assistances entail activities such as activities of daily life (ADL), cleaning or food delivery. Unplanned home care assistances are activities called upon by the older person when in need of urgent assistance, by use of a mobile safety alarm. The mobile safety alarm system used in home care settings is provided by nationally operating private companies, individually contracted by the municipalities. The mobile safety alarm device is linked to an alarm button in the form of a bracelet or necklace for the older person to press when an urgent need arises. When the alarm button is pressed, a non-medically trained operator from the private company responds via the installed hardware, and forwards information about the alarm to the municipal care personnel. If the older person asks for an ambulance directly, the operator can also alert the EMS. However, the mobile safety alarm is not intended for medical emergencies or life-threatening situations, in such cases older persons are urged to call 112 for EMS response. How to use, and when to use the mobile safety alarm is explained orally and in writing upon alarm device installation by the municipal care personnel.

The personnel in the emergency medical dispatch centre (EMDC) responding to the 112 calls in the municipality are all registered nurses (RNs). Ambulances are dispatched based on an assessment of the caller’s symptoms (Lindström et al., 2015). The ambulance services in the current municipality have at their disposal four emergency ambulances and one single staffed ambulance unit. Emergency ambulances are lifesaving units capable of advanced care and conveyance to hospital (Lindström et al., 2015). The single unit is not for conveyance, the main task being to shorten response time, to commence advanced care, or to assess diffuse conditions on-scene (Carlström & Fredén, 2017; Magnusson et al., 2016).

### Study participants

The municipal care personnel participating in the study include personnel working with planned home care assistances, and unplanned assistances related to mobile safety alarm calls (Table I). Henceforth both groups will be referred to as municipal care personnel. Swedish municipal care personnel usually comprise of assistant nurses with a training equivalent to upper secondary school level, ranging between 1.5–3 years, in addition to personnel without formal training (in Table I referred to as care assistant). Assistant nurse training in Sweden currently lacks nationally regulated requirements of competence and examination (SOU 2020:14). Municipal care personnel are not authorized to give any medication in emergency situations or carry out medical tasks other than cardiopulmonary rescue (CPR).

**Table I.** Characteristics of study participants

**Participant characteristics: all participants**				
Gender	Age range years (Mdn)	Assistant nurse (*n*)	Care assistant (*n*)	Work experience range years (Mdn)
Female	22–60 (47)	12	2	3–40 (21.5)
Male	44–58 (50)	5		17–31 (25)

**Participant characteristics: group interviews (personnel working with unplanned assistance)**				
Interview no.	Participants (*n*)	Gender (*n*)	Age range years	Work experience range years
1	3	Female (3)	22–56	4.5–37
2	3	Female (3)	44–53	3–27
3	2	Female (2)	47–59	20–40
4	2	Male (2)	46–58	17–27
5	3	Female (2) Male (1)	44–60	25–28
6	3	Female (1) Male (2)	47–57	26–31

**Participant characteristics: questionnaires (personnel working with planned assistance)**				
Participants (*n*)	Gender (*n*)	Age range years	Work experience range years	
3	Female (3)	46–52	10–20	

### Data collection

CIT allows for different data collection methods (Fridlund et al., 2017). In this study, data were collected through interviews and free text written questionnaires during October 2019 to March 2020. Group interviews were held with personnel providing unplanned assistances, strategically chosen, and appointed by the area manager in the municipality. In addition, a web-based questionnaire was launched to personnel providing planned assistances in one of 21 home care districts in the municipality. All municipal care personnel were given oral and written information about the study at workplace meetings, explicating voluntariness, and the right to end participation at any time without explanation or sanctions. A total of 16 personnel providing unplanned assistances volunteered for interview, and three personnel providing planned assistances returned the questionnaire.

The interviews were held in a secluded room at the participants’ workplace by the first author (A1) on six occasions. The interviews were individual, although they were conducted in small group constellations (Table I) as requested by the care personnel due to their working conditions and own choice. The group constellation enabled the participants to comment on and ask each other questions, thereby inducing ideas and memories which allowed more data to be collected from the participants (Morgan et al., 2013). If the interaction between the participants needed management due to loss of focus, the interviewer reset the focus to the individual interview. The interviews lasted between 72–84 minutes, during which time the participants were off alarm responding duty. Initiating questions regarded work-related opportunities and challenges. These were followed by the participant’s description of the critical incident (Table II). Follow-up questions were asked to further stimulate the descriptions of the situations, and to clarify experiences and actions. The same questions were used in the questionnaire, which was distributed as a link in an email along with written information about the study.

**Table II.** Initiating questions and examples of follow-up questions used in interviews and questionnaires

**Initiating questions:**
What is best about your job? What do you consider challenging about your job? Describe a situation when an older person needed ambulance. What happened?

**Examples of follow-up questions:**
How did you handle the situation? What happened when the ambulance personnel arrived? What were the consequences for you, and the older person? What do you consider positive and negative in the situation? How did you feel? In what way was the older person involved in decision-making in the situation? Why did you call for ambulance? Who made the decision to call for ambulance? How did you experience the collaboration with the ambulance personnel?

Due to exceptional and stressful working conditions within the SARS-CoV-2 pandemic (COVID-19), access to municipal care personnel was restrained, and further data collection by questionnaires was therefore cancelled. The three questionnaires already returned were included in analysis.

### Data analysis

The verbatim transcripts of the interviews and questionnaires were read repeatedly by the first author (AH), to become familiar with the content. In accordance with CIT analysis procedure (Fridlund et al., 2017), quotations indicating experiences or actions were inductively identified, and clustered separately into subcategories, stratified by their differences and similarities. In a similar way, the subcategories were clustered into categories, upon which overarching main areas were identified. An example of the analysis process is presented in Table III. The analysis process continuously oscillated between quotations and main areas to assure accurate descriptions. The result of the inductive analysis (Table IV) was discussed, challenged, redefined and validated by authors GÖ and MH. During the process, existing preunderstandings of the first author were challenged, and discussed to avoid perceptive interpretations to influence the result. The result was reviewed and approved by all authors.

**Table III.** Example of the analysis process

QUOTATONS INDICATING EXPERIENCES	SUBCATEGORY	CATEGORY	MAIN AREA
*It can take 40 minutes up to an hour before they arrive. And you understand that, they can’t really clone themselves either, they must prioritize (…) then, one has to wait. Even if they’re in pain. (A10, interview 4)* *She said herself that “I, this isn’t, this isn’t how I usually feel”. She wanted to go [to the ED] (…) She told the dispatcher too. But this woman [dispatcher] said “No. There’s no reason. I’m making an assessment that she doesn’t have to go [to the ED] by ambulance.” (A12, interview 5)*	Subjected to EMS priority	Conditions for EMS collaboration	Collaborative care
*They [the ambulance personnel] were so calm, and they were nice to her too, and to us too (…) It was nice in the room*. *(A7, interview 3)* *These times when it’s urgent, and they [the person] can’t talk, you know they’ve got dementia and they, they might not even want to go [to the ED], but there’s a need, then I think they [the dispatcher] should be able to (…) trust me. But it feels like they don’t, they don’t trust you. (A1, interview 1)*	Subjected to EMS attitude		

QUOTATIONS INDICATING ACTIONS			
*My colleague talked to 112, and then he brings the phone to the older man sitting there, who can barely breathe due to severe pain. You don’t talk that much then, to a person. Then 112 wants to talk! I just grabbed the phone “he can’t talk, he’s in so much pain, he can’t talk, send me an ambulance!”* *(A7, interview 3)* *No ambulance has arrived, so I call [112], and there she is in bed and can barely breathe and isn’t responding. So I call the ambulance “Where are you, it’s an emergency, you have to come!” (…) ”It’s going to be a while longer” they say. “Ok, but she’s unconscious and has a really hard time breathing, it won’t work, you have to hurry!” (A3, interview 1)*	Advocating the person	Representing the person	Adjusting to situational needs and EMS authority
*We help the ambulance personnel too, they usually leave [the scene]. Then we clean up, so the home is left ok*. *(A8, interview 3)* *We then understand that he’s going to the ED, so we start packing a bag, call his relative and say that “your dad is going to go to the ED, so you know.” (A5, interview 2)*	Handling practicalities

**Table IV.** Care personnel’s experiences of and actions in emergency situations involving the EMS, presented in main areas, categories, and subcategories

MAIN AREA	CATEGORY	SUBCATEGORY
EXPERIENCES		
Lifesaving competence	Emergency knowledge	Limited information
		Unsure decision-making
	A safeguarding companionship	Lifesaving responsibility
		Soothing presence
Collaborative care	Limited care options	Lack of care alternatives
		Lack of time
		Limited EMS resources
	Conditions for EMS collaboration	Subjected to EMS priority
		Subjected to EMS attitude
		Lack of routines

ACTIONS		
Adjusting to situational needs and EMS authority	Adapting to emergency needs	Assessing emergency signs
		Responding to care needs
	Representing the person	Advocating the person
		Handling practicalities
	Adapting to EMS leadership	Establishing EMS collaboration
		Complying with EMS directives

## Ethical considerations

Ethical approval was obtained by the Swedish Ethical Review Authority (Dnr: 2019–02027), and access to the personnel at the home care districts were granted by the area manager in the municipality, as well as the local manager of each home care district. Oral and written information was given before the start of each interview, and an informed consent was signed by each participant. Regarding the web-based survey, a returned questionnaire was accounted as valid consent to participate in the study. One local manager requested and received information about what questions would be asked during interviews but had no access to individual responses.

## Results

The critical incidents described by the care personnel emerged into the main areas *Lifesaving competence* and *Collaborative care* relating to experiences, and *Adjusting to situational needs and EMS authority* relating to actions (Table IV). The result will be presented as main areas and categories.

## Lifesaving competence

The municipal home care organization relies on the care personnel’s competence to identify emergency symptoms and make decisions of care in accordance with older persons’ needs. In practice, this means having sufficient knowledge to guide older persons in a wide range of emergencies, and for the care personnel to potentially save lives without having enough educational competence or organizational support. In a home care organization not adapted for handling acute illnesses and emergency situations, and in the absence of established emergency procedures or on-scene support, the care personnel become omitted to own lived experience and lifesaving competence to solve the situation. Lifesaving competence relies on having adequate *Emergency knowledge*, and an ability to enter into *A Safeguarding companionship*.

### Emergency knowledge

In emergency situations, care personnel shoulder the role of an emergency guide, the task being to guide and direct the older person through the situation. The care personnel describe how great trust is placed in their knowledge, and how the person seeks their guidance: “They want a recommendation. They want to hear our opinion” (A16). The care personnel describe how decisions on what to do are difficult to make. Not knowing the person, in addition to vague and diffuse symptoms complicate the ability to provide proper guidance. Sources of information within the organization that offer support in assessments consist of basic written documentation, and the possibility of consulting a college. Unfortunately, colleagues are described as difficult to reach. The care personnel thus have to make unsure decisions based on the limited information available: “How are you to know if it is an emergency or not? If it only, well affects the speech, and you don’t have any other symptoms” (A2). The care personnel describe how close relatives at times play an important role in providing information about a person’s general state of health. Relatives are additionally important in case of language barriers and stand in as interpreters.

For medical consultation, the care personnel must rely on regional phone support (1177, Swedish health care direct). Phone support, however, offers little scope for action: “They [1177] almost always say ’call an ambulance’. Because they don’t want to, well, it’s hard for them, they’re not on site” (A4). The care personnel express the need of having a registered nurse stationed within the home care organization, providing medical competence and counselling on-scene. Confirmation that unsure decisions to call for ambulance assistance are correct, is obtained by watching the ambulance personnel’s actions on-scene, and the medical treatment they provide. In contrast, the care personnel can feel incompetent if the person in need of care becomes alert and cheers up when the ambulance arrives. The lack of feed-back after involvement in emergency situations adds to the uncertainty of how to respond to perceived emergencies, preventing an evaluation of the situation.

### A safeguarding companionship

In emergency situations characterized by a person’s vulnerability, exposedness, and suffering, the care personnel enter into a safeguarding companionship. The companionship entails acknowledgement of suffering and acute care needs and brings responsibility for the person’s life. The care personnel describe how older persons often call on them rather than the EMS. This further emphasizes their responsibility, a responsibility that includes both a professional and a personal dimension. In their professional role, the care personnel are obliged by law to provide help in consideration of the person’s own will, regardless of the situation. The personal responsibility arises in the moment, emanating from a strong emotional response to the older person’s suffering. The two-folded responsibility is expressed as an ethical dilemma: “Personnel may not overrule someone who doesn’t want to call an ambulance. At the same time personnel are responsible for helping service users who are not feeling well” (A17). The personal responsibility carries a liability to solve the situation, which justifies going against a person’s will by persuasion or assumed decision-making. The personal responsibility remains until the liability can be transferred to another care provider. The care personnel describe how they try to provide calm and comfort while waiting:

[You] talk soothingly and say that the ambulance is on its way (…) That’s the only thing we can do, for now. Say that pain relief is on the way (…) show that you’re there, and that you at least know what it is about. And that you understand they’re in pain. And talk, talk. (A10)

The care personnel constitute relief in togetherness, and an assurance that help is on the way. The ability to create calm in emergencies demands experience, self-confidence, and a readiness for encountering serious conditions, which is described as lacking in young temporary personnel.

## Collaborative care

Emergency situations that occur within the municipal home care organization are dependent on collaborative care. Limited options of care require a well-functioning collaboration between personnel within, as well as between involved organizations. The lack of flexible care options, and well-established routines for collaboration in emergency situations risk jeopardizing older persons’ health. Collaborative care means the care personnel consider *Limited care options*, and to accept *Conditions for EMS collaboration*.

### Limited care options

The care personnel describe how options of care are very limited for older persons, revolving around transport to the emergency department (ED) or not. Many older persons are described as reluctant to go to the ED due to previous negative experiences related to long waiting times and inadequate care. The lack of care alternatives for low acuity conditions means having to send persons to the ED, despite knowing that pain and discomfort will be prolonged. Care personnel must decide if ED conveyance is necessary, and if so the mode of transportation required. The care personnel describe how ambulance transport is required in high acuity situations where advanced care is needed on-scene. In low acuity situations, transportation services are used. Transportation services, though, are not adapted to handle emergencies, and do not provide care even though they transport persons with acute illnesses. Transportation services therefore often come with long waiting times. When making the decision, the care personnel must balance the needs of the person, own workload, and time constraints, and at times this becomes a dilemma:

I do understand that you can’t call for an ambulance every time, but if you book the transportation service, then maybe it’ll come in, well a few hours. But what do I do for two hours? Maybe I have to respond to an alarm, it really doesn’t work. (A1)

To overcome long waiting times, the care personnel at times must turn to private options for transportation, such as relatives or taxis. Calling a taxi is described as the only option when the older person is perceived in need of rapid emergency response, but the perspective of what qualifies for dispatching an ambulance differs. There is also a financial issue to consider when an ambulance is not an available option. Private alternatives such as taxi require having the ability to pay up front, which affects the older person’s ability to access rapid care.

The care personnel describe how the organization does not differentiate between responses involving emergencies and responses involving standard home care, which become stressful. The lack of care alternatives, as well as the lack of time are frustrating, and affect the care personnel’s ability to respond in time to care needs:

We’re not an emergency service, but we do become one, in a sense, when we are the first ones on site (…) and then [name] alarms, who has home care 8 times a day, for toilet assistance (…) it feels frustrating. And we only have 40 minutes to get to him, but we won’t make that time, because we need to be here and now [handling an acute illness]. So, this with us not being an emergency service, it feels a bit ridiculous to say, because we actually are. (A4)

The lack of time also means that alarms must be prioritized by the care personnel themselves, leading to older persons having to wait for help. The knowledge that EMS resources are limited and to be used responsibly means that the care personnel themselves also make decisions about which emergencies legitimize calling for an ambulance. The care personnel describe how they, in the absence of emergency symptoms, can negate calling for an ambulance, so as not to burden the EMS.

### Conditions for EMS collaboration

The emergency medical services (EMS) assume authority in emergency situations, superior in their competence and medical resources. The care personnel thus become subjected to the EMS personnel’s professional knowledge, priority assessments, and attitude. When the EMS personnel assume control, the role of the care personnel shifts from being the one taking responsibility to the one following the lead. The shift is described as a welcome relief. However, the relief can also turn into coercion when the care personnel receive directions perceived to be ethically wrong, such as being directed to perform resuscitation on a deceased person when calling 112: “I do tell them that, well ’he is cold, I can’t, he’s not breathing, no pulse’. But still, I have to commence CPR” (A6). Still, the care personnel must always call 112 when an older person is deceased.

When calling 112, the care personnel must accept the ambulance dispatcher’s priority, and thus decisions regarding rapid, delayed, or denied ambulance care. The care personnel describe how ambulance dispatchers might disregard their competence, even though they are experienced personnel with extended knowledge about the acutely ill person. At times, the utilization of expensive EMS resources leads to having to defend the decision to call for an ambulance. The care personnel describe that their possibilities to influence ambulance dispatcher’s decisions are limited, even when the need for ambulance care is stressed. Being subjected to the priority setting of the ambulance dispatchers might mean having to wait for an ambulance, even when the older person is severely injured: “He had fractured both legs, and the ambulance had a lot to do that day, so it took another two hours before they arrived” (A6). Waiting for an ambulance is stressful, since remedy cannot be provided. The care personnel must also be prepared for worsening conditions.

When the ambulance arrives, the care personnel become subjected to the collaborative attitude of the ambulance personnel. The care personnel describe how the attitude of the ambulance personnel can differ in relation to the level of emergency. In acute emergencies, the ambulance personnel are perceived as acting highly professionally, showing competent leadership, and collaborative skills. This is in contrast to situations of low emergency when loss of interest in the older person may be displayed. The care personnel describe how they can feel frustrated and angry in situations when the ambulance personnel openly display little interest, ignorance, or disrespect towards them, and the older person. In contrast, collaboration in emergency situations can be experienced as positive and rewarding when the care personnel’s competence and participation in the situation are encouraged and valued. Positive collaborative experiences also relate to the ambulance personnel’s ability to be attentive and responsive to the older person’s needs: “It’s this feeling that she got help. And the treatment, that everybody treated her in a good way, there was a nice feeling in the room as well. And she was calm. She was safe” (A7). Attentiveness to needs promotes trust and well-being for all involved.

The lack of established routines guiding collaboration and communication in emergency situations affects the care personnel’s ability to adjust home care assistance to new conditions, for instance, when not knowing the outcome of the ambulance response. The care personnel further describe how the lack of communication entails risks related to the safety alarm system. Apart from requiring the older person to be able to handle the devices, the lack of established communication channels between the involved organizations prevents vital information being transferred:

Then we were a bit appalled, because if we had called the service user now, and asked her, well, “do we have to come?” (…) because sometimes we do that if there are a lot of alarms [waiting]. Then she would have replied, well “I have opened the door by myself, you don’t have to come”. And then it’s not sure if the ambulance ever would have come to her! Just because she couldn’t quite manage the phone. And it [the ambulance] wasn’t sent out. (A4)

The communication gap between the municipal home care organizations and the EMS constitutes a risk that jeopardizes older persons’ health, and consequences might never be detected.

The care personnel describe how care-dependent older persons’ lives become conditioned by the two organizations involved, between which the responsibility for the persons’ health and well-being oscillates and is unclear. The older persons are perceived to be bandied about:

There are far too many that have, are so ill due to multi-morbidity and on the border of being able to live at home, that we must send [to the ED]. And according to the hospital they are medically ready for discharge. Come home and cannot cope on their own (…) and then we must to send them back again. (A16)

The care personnel describe how the stressful working conditions characterizing home care further prevent detection of deteriorating health, which can result in unnecessary emergency situations requiring ambulance care.

## Adjusting to situational needs and EMS authority

Care personnel actions in emergency situations revolve around adjusting actions according to the different dynamic and emerging requirements. The requirements directing actions are related to the perceived emergency needs, duty of work, and EMS authority. Based on the older person’s needs, the care personnel initiate EMS collaboration as the older person’s representative. Subsequent actions are governed by EMS leadership and directives that encourage or restrain care actions. Adjusting actions to surrounding requirements leads to *Adapting to emergency needs, Representing the person*, and *Adapting to EMS leadership*.

### Adapting to emergency needs

The care personnel search for and assess existing emergency symptoms and needs. The assessment of emergency signs involves listening to the older person, looking for visible injuries and symptoms of illness. When symptoms are diffuse, further information is gathered by reading all available documentation, by calling colleagues, the person’s relatives, or 1177 to enhance understanding of the situation. After assessing the emergency needs the care personnel suggest a measure. In dialogue with the person, consent to the suggestion is negotiated:

We were communicating for quite some time with her before we called (…) we talked with her for 20 minutes for sure before we took a [decision] “shouldn’t we call anyway?” We never call [112] unless the person agrees to it. (A2).

If symptoms are vague, much weight is placed on the older person’s own experience of the situation, which guides the care personnel’s actions. In perceived time critical illnesses, such as symptoms involving the heart, visible injuries, severe bleeding, unconsciousness, or other rapid health deteriorations, the care personnel take responsibility for decision-making and call an ambulance: “She had become very ill in just a couple of hours (…) I talked to her and she couldn’t tell me what had happened (…) I called 112 directly” (A18). The care personnel respond to shifting care needs presented in the situation. When the acute emergency need is met, the care actions adapt to a waiting phase. During this phase the care actions serve to provide comfort or alleviate discomfort, and extend to others involved in the situation such as the person’s partner, relatives, or at times colleagues. The care personnel provide help and support through caring actions, by talking, listening, and by just being present.

### Representing the person

Due to the vulnerable condition of the suffering person, the care personnel take on the role as the person’s representative and mouthpiece. Being the older person’s representative means acting on behalf of the older person and involves advocating actions in the interaction with the EMS, in addition to being a physical extension in handling the practical matters the situation brings about. Examples of the care personnel’s advocating actions are giving suggestions to improve situational care or providing EMS personnel with vital information about the older person. Advocating also implies prompting ambulance responses, or questioning EMS decisions: ”What I do then, I question this [ambulance dispatcher], saying ’do you take responsibility that this will be ok, him going by transportation service?’ After that she sent an ambulance, she did. Because I can’t, take that responsibility” (A13). Practical actions involve preparing for ambulance conveyance and hospital stay. The care personnel pack personal belongings, cellphone, and keys, and make calls to forward information about the new situation to relatives, colleagues, or the ED. The care personnel also clean up to restore order in the person’s home after the emergency response.

### Adapting to EMS leadership

The care personnel initiate contact and collaboration with the emergency medical services, and as a result adapt actions to EMS leadership and directives. During the 112 call the care personnel constitute a link between the ambulance dispatcher and the older person, and the care personnel perform actions through dispatcher guidance: “And then we have them on speakerphone the whole time, so they, well, guide us. So, we just turned her over and start CPR” (A5). When an ambulance is dispatched, the care personnel prepare for the ambulance personnel by opening doors and obtaining current medicine-lists to enable rapid and smooth access to the person. On ambulance arrival, the care personnel meet up and provide the ambulance personnel situational information. Subsequent actions comply with the directives of the ambulance personnel: “If they need help, they say so (…) So they’re very clear, I think, with what they want, either we go or we stay” (A4). Adapting to EMS leadership means the care personnel become a resource for EMS personnel to use at will.

## Discussion

The aim of this study was to describe municipal care personnel’s experiences of and actions in situations when older persons need emergency medical services at home. The result reveals areas that involve challenges significantly affecting the care personnel’s possibility to respond to older persons’ unique emergency needs, and to provide safe care in emergency situations. This runs counter to the vision of current Swedish political health care reform which promotes a person-centred sustainable health care system, redirecting the health care setting from hospital to out of hospital care (SOU 2020:19). The political reform is based on the internationally adopted Agenda 2030 (UN General Assembly, 2015), and considered to promote an equal, accessible, integrated and efficient care that benefits vulnerable groups in society. In practice, this means that care-dependent older persons are to be provided qualitative care characterized by accessibility and security based on individual needs. The study is therefore an important contribution to the ongoing development towards qualitative out of hospital care, since the result reveals deficiencies that must be addressed if the governmental goals are to be achieved. This study provides knowledge that increases understanding of the challenges at different levels related to ageing in place and ageing well. This is of significance both nationally and internationally. The following discussion focuses on these challenges from a bottom-up approach containing three levels: the personal, the collaborative and the organizational level. The result also reveals dimensions of older persons’ participation in care, which will be addressed from a lifeworld perspective in relation to the aforementioned levels.

### Challenges at the personal level — managing emergency situations with limited knowledge and support

The result displays how care personnel are responsible for providing adequate care in emergency situations without sufficient knowledge, medical or organizational support. Since the municipal home care organization does not recognize emergency care as part of the services, the education level of personnel, medical support, and options of care are not adapted accordingly. Thus, care personnel become left on their own to solve emergency situations. The challenge of solving emergency situations without appropriate knowledge, support or flexible care alternatives becomes accentuated in relation to the professional responsibility stated in laws and policies requiring care personnel to provide safe care according to individual needs. Furthermore, older persons’ acute illnesses are described as challenging to assess. Acute conditions are often displayed in low acuity, and symptoms might be displayed atypically or masked due to polypharmacy or chronic diseases (Durant & Fahimi, 2012; Horibata & Takemura, 2015; Shenvi & Platts-Mills, 2019). Even highly educated RNs in the Swedish EMS describe that assessing patients involves great responsibility, paved with feelings of uncertainty, loneliness, and fear of making harmful decisions (Höglund et al., 2019; Lederman et al., 2019). Despite these well-documented challenges, the home care organization places assessing responsibility on assistant nurses and care assistants without providing appropriate medical support, which risks jeopardizing older persons’ health and well-being. Competence has also been found to be higher among care personnel in nursing homes which usually include RNs, compared to care personnel in home care where RNs are lacking (Bing-Jonsson et al., 2016). A higher educational level (bachelor’s degree) among personnel in care contexts has been found to improve health outcomes and decrease patient mortality (Aiken et al., 2014), which emphasizes the need for RNs to be stationed within municipal home care to fulfill requirements of safe care. The challenge related to limited care alternatives also forces care personnel to “fight” for ambulances, or find solutions outside the organization, for example, turning to relatives or public transportation, such as taxi, for transport to hospital to receive urgent care. The necessity for care providers to find solutions outside the organization, or bend the rules to meet complex care needs is also pointed out in the study by Kuluski et al. (2017), who describe a need of having to ’work the system’ to provide appropriate care to persons in need of both social and health care. The current lack of well-functioning support structures within the municipal organization needs to be urgently addressed if care personnel are to be able to provide person-centred and safe care.

### Challenges at the collaborative level — dependence on inter-professional collaboration

Care personnel must conform to and accept the collaborative conditions provided by the EMS. Thus, the personnel and the older persons are in the hands of the EMS organization, and consequently the priorities and attitude of EMS personnel. The hierarchy existing in health care thus becomes evident, where the care personnel in the municipal organization are subordinate in relation to the regional organization and the EMS in charge of all emergency resources. Due to the ongoing movement towards inter-professional collaboration, researchers call for health care systems to address the issue of power in collaborative constellations, and how it impacts clinical practice and care goals (Cohen Konrad et al., 2019). The dependence on inter-organizational collaboration in emergency situations, and the lack of structured collaboration between the EMS and the municipal home care organization put older persons’ well-being at risk. Research on inter-professional collaboration stresses the need for clarified roles, shared goals, respectful communication, and recognition of interdependence for collaboration to be successful in the care of older persons (Montano, 2019). The lack of clearly defined roles for care personnel within municipal home care have been pointed out in previous research (Bing-Jonsson et al., 2016). The result of this study further advances the need for role clarification in relation to emergency situations occurring within municipal home care.

The result also displays how care personnel might feel distrusted by the EMS, and at times encounter negative attitudes. Trust is vital for successful collaboration, and all personnel involved need to have a positive attitude towards collaboration and acknowledge that the collaboration will positively affect care (Bookey‐Bassett et al., 2017). In this study, negative attitudes were experienced related to situations characterized by diffuse symptoms and low acuity. In a previous study, ambulance nurses were found to struggle to display interest in persons who were not perceived as being in need of emergency care, which could lead to the nurses being frustrated and rude (Barrientos & Holmberg, 2018). The need for developing a structured collaboration between organizations to assure safe and qualitative care is urgent, where level of acuity does not determine quality of care.

### Challenges at the organizational level — an organizational structure not adapted to older persons*’* emergency needs

Care personnel are to provide person-centred care in an organization that offers few care alternatives when emergencies occur. The option in hand is provided by the regional health care organization and limited to phone support or ambulance response resulting in hospital and emergency department (ED) conveyance. In lack of adequate care alternatives, the care personnel cannot offer a solution adapted to the person’s unique emergency need. Thus, the older person is forced to accept the suggested option of care, in line with previous research (Jarling et al., 2018; Ploeg et al., 2019). It is also well known that ED conveyance of older persons risks negatively affecting their health outcome, involving long waiting times (Bunn et al., 2019), and increased risks of infection, delirium, and pressure ulcer development (Dugaret et al., 2014; Hwang & Morrison, 2007). Such negative experiences of previous ED admittances make older persons reluctant to accept ED conveyance (Smith-Carrier et al., 2017; Vicente et al., 2013).

An important aspect concerning the care options being provided by regional health care is that older persons must compete for EMS resources with the general population, which raises the question of accessible and equitable care. In a previous study we found that older persons receive lower priorities in comparison to the general population when in need of ambulance care (Hjalmarsson et al., 2020), and the assigned priority has been found to be too low in relation to the acuity of the condition in several studies (Benjamin et al., 2018; Brown et al., 2019; Hoyle et al., 2020). Age discrimination is also prominent in a study by Wiel et al. (2017), where older age was found to determine less and shorter duration of emergency care for patients with cardiac arrest. A recent study reports an increasing trend of non-conveyance of older persons (Forsgärde et al., 2020), emphasizing that the arena for advanced emergency care is shifting from the hospital to the home. This increases the need for the municipal home care organization to adapt services to also include readiness for emergencies and provide care options that align with the shifting emergency needs of care-dependent older persons.

Welfare technology in the form of mobile safety alarms is an important component of municipal home care services. The purpose of welfare technology, such as mobile safety alarms, is to promote health and safety (National Board of Health and Welfare, 2020). Older persons also place great trust in the alarms, describing them as essential for feeling secure at home (Melander‐Wikman et al., 2008). Even though the alarm is not intended for acute illnesses, the care personnel’s descriptions of emergency situations tell a different story. The result of this study reveals that care-dependent older persons use the mobile safety alarm for all sorts of emergencies, including life-threatening situations characterized by conditions such as severe bleeding, fractures, and chest-pain. The care personnel describe serious deficits in the mobile safety alarm system, relating to practical use and inter-organizational collaboration. Firstly, the technology requires that the users know how to handle the device. Secondly, stressful situations have been found to affect cognitive ability, and the user forgets to press the alarm (Stokke, 2017). Finally, setting off the alarm triggers a chain reaction that is currently unstable involving three parties: the operator in the private company providing the alarm system, the municipal care personnel, and the EMS. The result of this study reveals communication breakdowns between the parties, which constitute a severe risk of an emergency response failure jeopardizing older persons’ health. Research points to the lack of a designated person with overarching responsibility as an important obstacle for effective collaborative communication in the care of frail older persons (Hansson et al., 2018), this is also visible in the present study. Effective communication channels between involved parties, and a clear understanding of who carries the overarching responsibility for the emergency response are vital to ensure older persons’ safety when using the mobile safety alarm that involves the EMS.

### Implications for participation in care from a lifeworld perspective

The results of this study indicate important dimensions of participation that need to be highlighted and discussed from a lifeworld perspective. The care personnel’s lived experience of emergency situations reveals aspects of care-dependent older persons’ lifeworld, where participation in care becomes accentuated due to the extreme character of the care situation. Participation in care from a lifeworld perspective means involving the ’whole person’ in care situations, and understanding that well-being is an existential experience (Dahlberg et al., 2009). When viewing the result from a lifeworld perspective, dimensions of participation are found relating to the personal, collaborative and organizational levels. The different dimensions reveal matters that both impede and promote participation in emergency situations.

When encountering the older person, the care personnel enter into a companionship characterized by a recognition of a suffering human being. This companionship means entering into the older person’s lifeworld, providing a soothing presence in togetherness with the ability to ease suffering and existential loneliness, described in literature as vital dimensions of a humanizing care practice that centres the person (Todres et al., 2009). Recognition of care-dependent persons’ lifeworld promotes their feelings of being safe, supported, comforted and in control (Todres et al., 2014). The companionship indicates the care personnel’s engagement, and commitment to respond in the best way possible to all needs displayed in the situation. When not being able to visually assess the acuteness of the situation, much emphasis is placed on the older person’s lived experience of the situation. By listening to the older person’s story, the care personnel become open to the person’s lifeworld (Dahlberg et al., 2009), allowing for a deepened understanding of the person’s values and beliefs which promotes participation in care. The care personnel’s use of listening as the main source of information also engenders trust, which older persons view as a key aspect for experiencing participation (Soares et al., 2019). However, the inability of care personnel to acknowledge the older person’s lifeworld might lead to distrust, impeding participation and risking violating human dignity (Todres et al., 2014).

By giving emphasis to the older person’s experience of the situation, limitations and abilities in the person’s lifeworld are revealed that guide the care personnel’s actions. In acknowledgement of the older person’s lifeworld, in the moment presenting itself conditioned by acute suffering and vulnerability, the care personnel implicitly shoulder a safeguarding and a representative role. The care personnel’s actions as the older person’s representative empower the older person, promoting agency and active participation in care in line with a humanizing care upholding human dignity (Todres et al., 2009). Dahlberg et al. (2009) refer to well-being as a person’s ability to experience peace, and the care personnel’s assuming of a representative role can be viewed as a possibility for the older person to experience peace, and thus well-being, despite being in a state of acute suffering. The transferred participation also enables the personnel to safeguard the person’s best interests even if the older person is not present, extending participation beyond the situation. Such advocating actions uphold the older person’s safety, and dignity in both life and death.

The care personnel’s enactment of the transferred participation faces challenges in collaboration with the EMS. The care personnel’s experiences reveal how their ability to act as the older person’s representative can be both impeded and promoted by EMS personnel, significantly affecting participation agency. Descriptions of positive collaboration entail positive attitudes by EMS personnel, which encourage the care personnel to participate in the care situation and contribute with knowledge and suggestions for care. Such collaboration can be considered as significantly promoting older persons’ participation in care enacted by the care personnel. In contrast, the negative attitudes of the EMS in addition to protocol privileged emergency assessments, effectively impedes participation in care. Negative experiences of EMS collaboration reveal ignorance of the older person’s lifeworld. Such ignorance negatively affects well-being for both care personnel and the older person, and might render feelings of being abandoned, unimportant and unworthy of care, thus infringing human dignity (Todres et al., 2014).

From a welfare perspective, participation in care is a fundamental aspect of ageing in place and ageing well. However, participation in care requires that older people are able to participate in care, especially if ageing well is dependent on context specific conditions which emphasize the municipalities’ responsibility for its ageing population (Ehni et al., 2018). The current structure of prehospital emergency care for care-dependent older persons, and the very limited emergency care options available poses a challenge for participation, and the ability to influence care. Despite legal governance demanding participation in care with emphasis on care-dependent persons’ preferences and needs, the result clearly reveals how the current organizational health care structure endorses a ’one size fits all’ care which effectively counteracts person-centred care practices. If the goal of care is to promote well-being it must be characterized by flexibility rather than fixed solutions, which requires a supportive organizational structure (Dahlberg et al., 2009). The required preconditions to provide care according to care-dependent older person’s preferences in emergency situations are currently lacking, restricting their ability to participate in and influence care.

## Methodological considerations

Flanagan (1954) emphasizes the importance of reporting all possible biases introduced during CIT study procedure. A sufficient number of critical incidents for analysis is important in order to achieve a comprehensive understanding. A limitation is, therefore, the fact that the online questionnaire was cancelled due to the COVID-19 pandemic, thus limiting the number of described critical incidents for analysis. The final number of critical incidents described in full was 63, in addition to general descriptions and experiences of emergency situations involving care-dependent older persons. According to Flanagan (1954) a minimum of 50 critical incidents might be considered sufficient for analysis, and due to the unsure circumstances related to the pandemic the authors agreed that the rich data already collected was to be considered enough. Another weakness in the questionnaire is the lack of elaboration in the written descriptions. In contrast, the situations described during the interviews were very well elaborated on and rich in detail, and therefore considered to make up for the loss of detail in the three written descriptions.

An issue of dependability refers to the participants being representative enough to answer the research question. The care personnel were strategically selected, and the sample consisted of all personnel but one working with unplanned assistance in the municipal organization at the time, together with three personnel working with planned assistance. The purposive sampling contained only one criterion for inclusion which was experience of an emergency leading to EMS involvement. Such sampling might lead to questions about sufficient variation among participants. However, as can be viewed in Table I, the participant characteristics display diversity regarding age, educational background, and work experience. In addition, both females and males are represented despite the home care organization being a female-dominated workplace. From a lifeworld perspective, each participant has an inherently unique lived experience that cannot be compared to or interchanged with someone else’s lived experience. A considerable limitation due to the pandemic and the cancellation of the questionnaire, is that home care personnel providing planned assistances were not represented sufficiently enough which might have affected the result.

The individual interviews conducted in small groups were not part of an intentional study design, but the result of existing working conditions and the care personnel’s own choice. The interview method however provided a combination of both individual in-depth descriptions of critical incidents, as well as general experiences of emergency situations arising from the spontaneous discussions. This resulted in richer data. One limitation of the individual interview being conducted in group is that the participants can be reluctant to speak freely in the presence of colleagues. However, the interaction between the participants was found to favour memory recall since they reminded each other of details or other situations to tell about, and they also discussed, shared, and compared the situations amongst themselves which contributed significantly to the elucidation of actions and experiences. This unexpected co-construction of interviews was considered a strength, described as an advantage of dyadic interviewing (Morgan et al., 2013). The spontaneous group discussions could also result in loss of focus on the topic. In such cases, the interviewer reset the focus to the individual interview. Another limitation is how well the participants remember the situation, since memory gaps increase the risk of the truth being manipulated (Fridlund et al., 2017). However, the participants described situations that had emotionally affected them, and some of the situations related directly to life and death situations which can be considered to substantially enhance memory.

Confirmability and researcher bias is another important issue of CIT. The interviewer’s wording might influence participants by encouraging them to address certain aspects of the situations, increasing the risk of them telling what the interviewing researcher wants to hear. By using open-ended questions, and letting the participants speak as freely as possible, the bias might be considered to have been reduced. During the analysis process, the results were additionally continuously discussed, and preunderstandings were challenged.

An issue of credibility is the study’s implicit investigation of older persons’ participation in emergency situations at home, and that the participants did not address participation per se. However, ’actions speak louder than words’, and while not asking for perceptions of older persons’ participation in the situations, more dimensions of participation could emerge. The rich data, although with the limitations mentioned above, clearly illustrates older persons’ ability to participate, and influence care in emergency situations.

The results of the study are transferrable to similar contexts in Sweden and neighbouring countries that share similar welfare structures. Limitations exist in transferring the results to a wider international level due to the structural differences of health and social care organizations within countries. However, the knowledge of this study deepens the understanding of challenges related to ageing in place and older persons’ acute care needs, which might be of international relevance despite cultural differences between countries.

## Conclusion and implications

The lack of organizational support, flexible care alternatives, and structured inter-organizational collaboration jeopardize care-dependent older persons’ health and ability to influence care when emergency situations occur at home. Municipal home care service organizations need to acknowledge the occurrence of emergency situations as a natural part of everyday life for older persons, implying organizational readiness to meet the shifting emergency needs of persons living in dependence at home. Municipal care personnel’s actions as the older person’s representative support human agency, allowing the older person to become an active participant in care despite being in a situation of acute suffering. The transferred participation emphasizes the need for the EMS to actively involve and collaborate with municipal care personnel in emergency situations, thus supporting participation in care. The EMS need to incorporate the whole life situation in assessments, emphasizing the need for including the knowledge and perspectives of the care personnel. However, more research is needed focusing on informal caregivers, such as family members, friends or neighbours, and their role in prehospital emergency care for older persons which is also of significance in developing ageing in place. The present study underlines the importance of further developing welfare policies that facilitate and regulate the inter-organizational responsibilities of health and social care to favour care-dependent older persons’ participation in care, thus promoting health and well-being in older age.
